# Controlling the proximity effect in a Co/Nb multilayer: the properties of electronic transport

**DOI:** 10.3762/bjnano.11.118

**Published:** 2020-09-07

**Authors:** Sergey Bakurskiy, Mikhail Kupriyanov, Nikolay V Klenov, Igor Soloviev, Andrey Schegolev, Roman Morari, Yury Khaydukov, Anatoli S Sidorenko

**Affiliations:** 1 Lomonosov Moscow State University, Skobeltsyn Institute of Nuclear Physics, Moscow, 119991, Russia; 2 Lomonosov Moscow State University, Physics Department, Moscow, 119991, Russia; 3 Moscow Technical University of Communication and Informatics (MTUCI), 111024 Moscow, Russia; 4 Lobachevsky State University of Nizhny Novgorod, Nizhny Novgorod 603950, Russia; 5 Moscow Institute of Physics and Technology, State University, Dolgoprudny, Moscow Region 141700, Russia; 6 Institute of Electronic Engineering and Nanotechnologies ASM, MD2028 Kishinev, Moldova; 7 Max-Planck-Institut für Festkörperforschung, Heisenbergstraße 1, D-70569 Stuttgart, Germany; 8 Laboratory of Functional Nanostructures, Orel State University named after I.S. Turgenev, 302026, Orel, Russia

**Keywords:** cryogenic computing, spin-valve, superconducting neural network, superconducting spintronics

## Abstract

We present both theoretical and experimental investigations of the proximity effect in a stack-like superconductor/ferromagnetic (S/F) superlattice, where ferromagnetic layers with different thicknesses and coercive fields are made of Co. Calculations based on the Usadel equations allow us to find the conditions at which switching from the parallel to the antiparallel alignment of the neighboring F-layers leads to a significant change of the superconducting order parameter in superconductive thin films. We experimentally study the transport properties of a lithographically patterned Nb/Co multilayer. We observe that the resistive transition of the multilayer structure has multiple steps, which we attribute to the transition of individual superconductive layers with the critical temperature, *T*_c_, depending on the local magnetization orientation of the neighboring F-layers. We argue that such superlattices can be used as tunable kinetic inductors designed for artificial neural networks representing the information in a “current domain”.

## Introduction

Multilayer superconductor/ferromagnetic (S/F) heterostructures can be used for construction of tunable cryoelectronic elements, such as switches, Josephson junctions and inductors [1–8]. Here, we present theoretical and experimental investigations of an S/F “stranded wire” with a controllable proximity effect. The wire is composed of ferromagnetic (F) layers separated by thin superconducting layers, in which the superconducting order parameter is maintained due to the proximity to a thick superconducting bank (S-bank). Switching from the antiparallel (AP) to the parallel (P) alignment of neighboring F1 and F2 layers leads to a significant enhancement of the effective exchange field in this artificial ferromagnet. Previously, the properties of [Co(1.5 nm)/Nb(8 nm)/Co(2.5 nm)/Nb(8 nm)]_6_ multilayer structures for cryogenic memory applications were studied using polarized neutron scattering and magnetometry techniques [9]. In particular, the parameter regions where the aforementioned switching between the P and AP orientations of the F1 and F2 layers is possible were found experimentally.

In this work, we perform theoretical and experimental analyses of electronic properties of Nb/Co multilayers with different F1 and F2 thicknesses and several stacking periods. It is demonstrated that the magnetization switching results in modulation of superconductivity in the superlattice with a corresponding change in the kinetic inductance of the superconducting parts of the wire core, due to the inverse proximity effect. We argue that this effect facilitates new possibilities for the development of tunable superconducting electronic components. For example, the considered “stranded wire” can be readily applied in a synaptic connection for a superconducting artificial neural network (ANN), where the information is represented in a “current domain” [10–21].

The paper is organized as follows. In the next section, we highlight how the proximity effect modulates hybrid S/F structures (the most interesting of the applications discussed), present the model and methods used in the theoretical research, and discuss the obtained results. In the “Experimental results” section, we analyze the transport measurements of the manufactured samples. At the end, we discuss possible applications of the results for the implementation of superconducting synapses and give a conclusion.

## Results

### Model and theoretical results

Contrary to traditional semiconductor basic elements (transistors), tunable kinetic inductors (TKIs), as well as nonlinear elements (Josephson junctions), are not fabricated in a substrate. This allows for 3D topology benefits, which are especially important for deep ANNs. The F1/s/F2/s superlattice, in which the thick S-bank acts as the source of induced superconductivity, is the simplest model of the 3D structure. Let us consider the applications that are possible due to the control over the order parameter in thin superconductor layers (s-layers) in such a structure.

**Figure 1:**
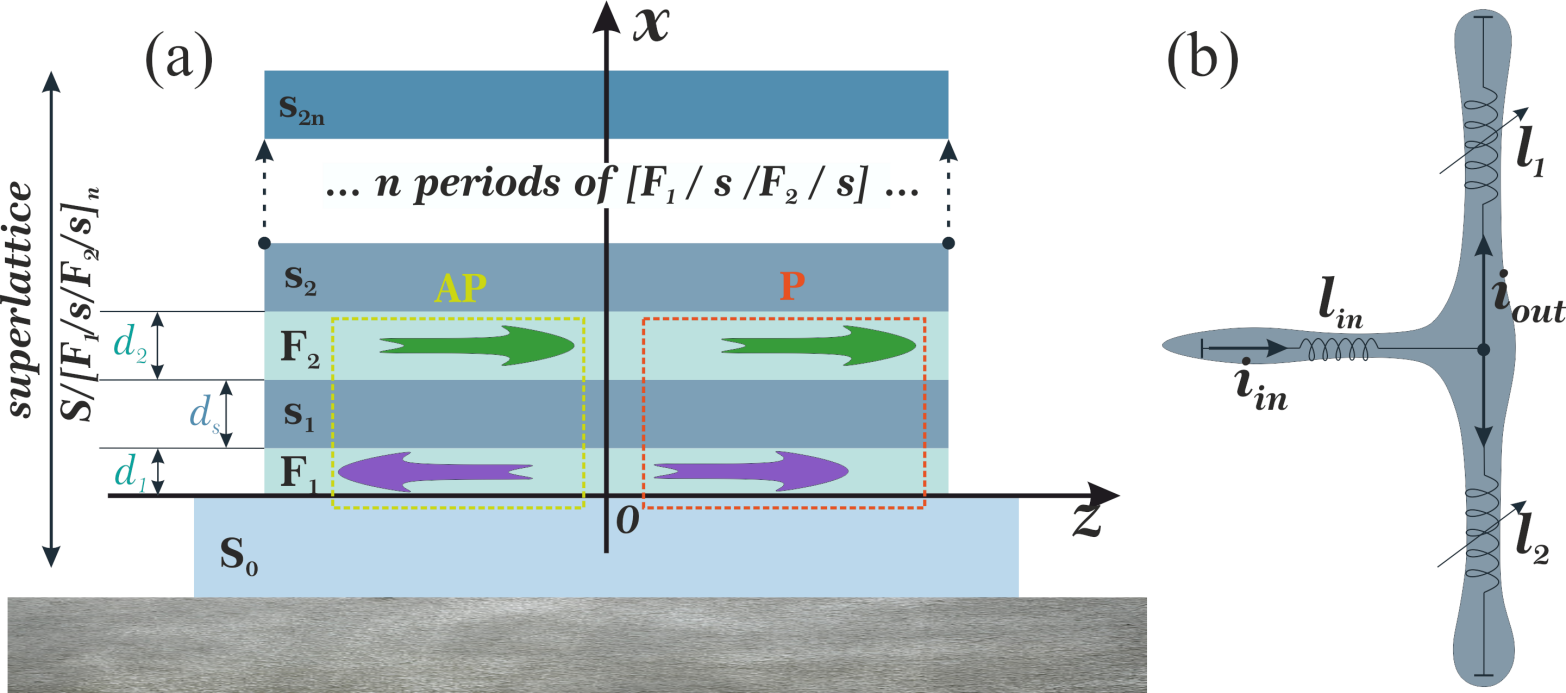
(a) Sketch of the investigated multilayer Co(1.5 nm)/Nb(6 nm)/Co(2.5 nm)/Nb(6 nm) structure. (b) The simplest splitter model based on this TKI.

The simplest cell for the current flow control using the TKI is a splitter. The input current, *i*_in_, induced in the input inductance, *l*_in_, splits between the two TKI elements. Figure 1b presents the principal scheme of a synaptic element in a superconducting ANN (with TKI elements instead of Josephson junctions, which were used previously [20]).

The synapse modulates the “weight” of an arriving signal, which corresponds to the input current. The transfer function of this current transformer can be described as follows:

[1]
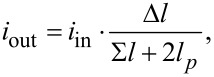


where *i*_in_ and *i*_out_ stand for the normalized input/output current, respectively, Δ*l* = *l*_1_ − *l*_2_ and Σ*l* = *l*_1_ + *l*_2_; *l*_1_, *l*_2_ are the normalized inductance values of two TKIs, and *l_p_* is the stray geometric inductance of a splitter branch. For this device to function properly, it is critically important to find conditions in which the kinetic inductance changes significantly due to the controlled proximity effect in the S/F structure.

To test the concept of the magnetically tunable kinetic inductor, we calculated the superconducting order parameter in S/[F1/s/F2/s]*_n_* superlattices, as presented in Figure 1.

We studied the proximity effect and electronic transport in the multilayer hybrid structures in the frame of Usadel equations [22],

[2]
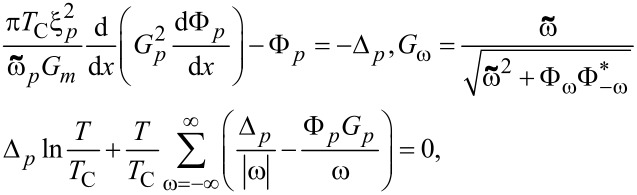


using the Kupriyanov–Lukichev boundary conditions [23–24],

[3]
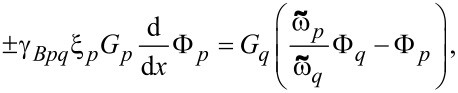


at the S/F interfaces. Here, *G_p_*,*_q_* and Φ*_p_*,*_q_* are normal and anomalous Green’s functions, respectively, and ω = π*T*(2*n* + 1) is the Matsubara frequency. In addition, 
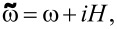
 where *H* is the exchange energy in the F-layer, *p* and *q* are indexes that denote the materials, ξ*p* is the coherence length, γ*_Bpq_* = *R_BA_*/ρ*_p_*ξ*_p_* is the interface parameter, in which *R_BA_* is the resistance per square of the interface, and ρ*_p_* is the resistivity of the material at the p-side of the boundary. Note that the boundary conditions at the S/F interface are written from both sides, leading to two independent parameters, γ_BSF_ and γ_BFS_. The ratio between these parameters, γ = ρ*_S_*ξ*_S_*/ρ*_F_*ξ*_F_*, is a suitable parameter to understand the physics of the system, since it depends only on the material properties.

In our calculations we put the origin of the *x* axis at the free interface of the bulk semiconductor electrode, with thickness *L*_S_ = 10ξ*_S_*. In addition, we considered the proximity effect of an artificial ferromagnetic material (AFM), consisting of alternating thin superconducting (*L*_S_ = 1ξ*_S_*) and ferromagnetic layers, with an exchange energy of *H* = 10*T*_C_. In an AFM, every odd-numbered ferromagnetic layer has a thickness of *L_F_*_1_ = 0.15ξ*_S_*, while every even-numbered ferromagnetic layer has a thickness of *L_F_*_2_ = 0.1ξ*_S_*. We assume that the diffusive coherence length of the superconducting and ferromagnetic materials are the same; however, the relative resistivity values can differ. The numerical solution of the boundary problem (Equation 2, Equation 3) provides the required spatial distribution of the pair potential, Δ(*x*), as well as the anomalous, Φ(*x*), and normal, *G*(*x*), Green’s functions at a given temperature.

**Figure 2:**
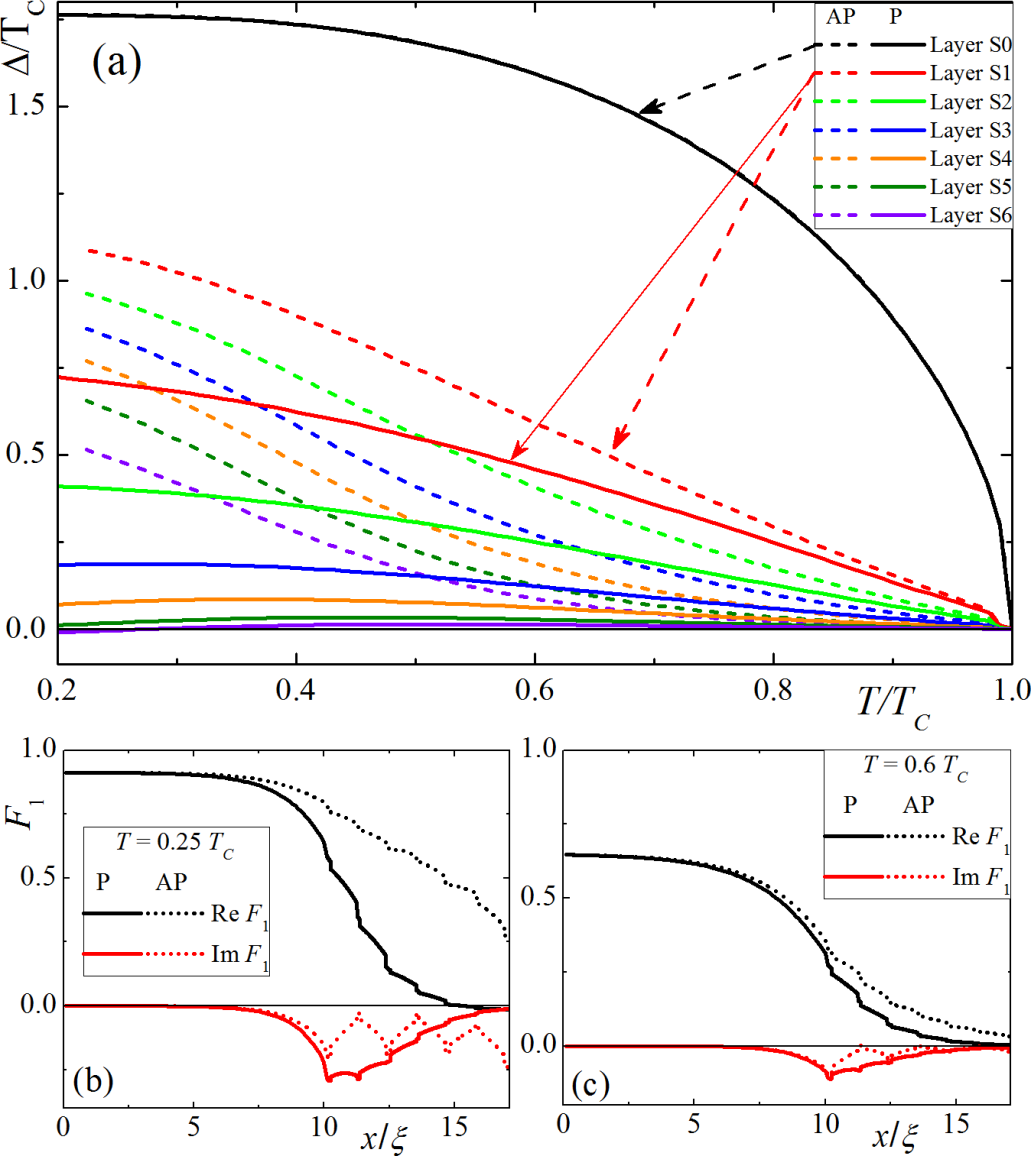
(a) Pair amplitude, Δ, in a S(F1sF2s)_x3_F1 structure in different superconducting layers as a function of temperature, *T*, for parallel (solid lines) and antiparallel (dashed lines) magnetization orientations, considering γ = 1. (b,c) Spatial distributions of the anomalous Green’s function, *F*_1_(*x*), for parallel (solid lines) and antiparallel (dashed lines) magnetization orientations at different temperature values: *T* = 0.25*T*_C_ (b) and *T* = 0.6*T*_C_ (c)_._

We found that the behavior of the system significantly depends on the relative resistivity values and coherence lengths of a chosen material. When the ferromagnetic metal and the superconductor have the same resistivity and diffusion coefficients (i.e., for γ = 1), the pair potential in the whole structure grows evenly with the temperature decrease (Figure 2a). The main source of the superconductivity is the bulk semiconductor layer, while the thin s-layers only slightly support the pairing amplitude coming from the source. Figure 2b shows the spatial distributions of the anomalous Green’s function


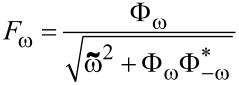


at the first (*n* = 0) Matsubara frequency, *F*_1_(*x*), for parallel (solid lines) and antiparallel (dashed lines) magnetization orientations at low, *T* = 0.25*T*_C_ (panel b), and high, *T* = 0.6*T*_C_ (panel c), temperature values. The real part of *F*_1_(*x*) decreases inside the AFM almost exponentially, with a small step-like modulation in thin superconducting layers. In the antiparallel case, the real part of the functions decreases slowly with an increase in *x*. At the same time, the imaginary part behaves differently for parallel (P) and antiparallel (AP) configurations. In the AP case, the imaginary part oscillates, returning to almost zero after every second layer. In the P case, the imaginary part decreases almost exponentially. However, this decrease is slower than the decrease observed for the real part of the function, increasing the possibility of a 0–π transition.

**Figure 3:**
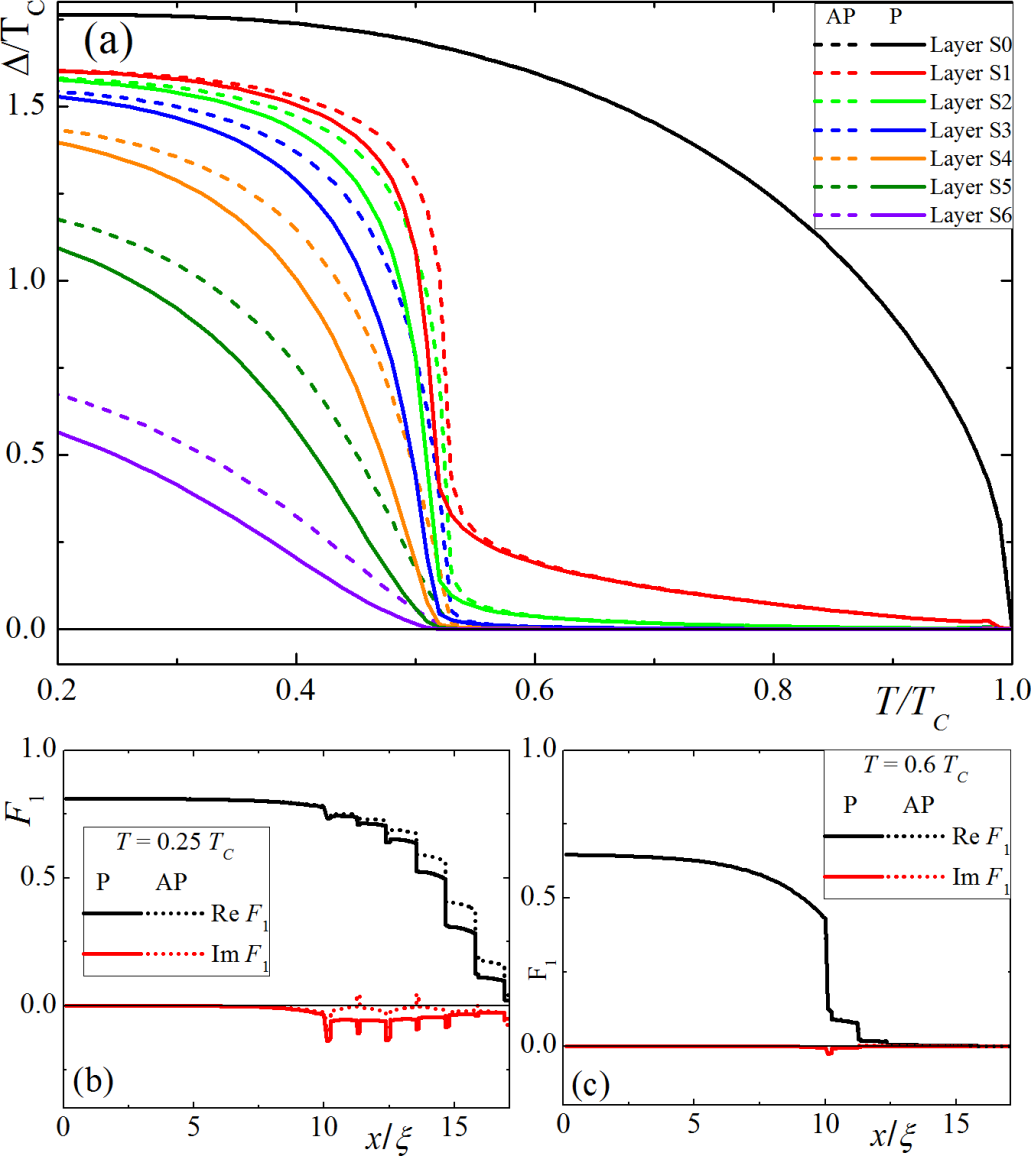
(a) Pair amplitude, Δ, in a S(F1sF2s)_x3_F1 structure in different superconducting layers as a function of temperature, *T*, for parallel (solid lines) and antiparallel (dashed lines) magnetization orientations, considering γ = 0.1. (b,c) Spatial distributions of the anomalous Green’s function, *F*, for parallel (solid lines) and antiparallel (dashed lines) magnetization orientations at different temperature values: *T* = 0.4*T*_C_ (b) and *T* = 0.6*T*_C_ (c)_._

However, the proximity effect properties are completely different if the resistivity of the superconductor is significantly smaller than that of the ferromagnetic material (γ = 0.1*).* In this case, thin s-layers are protected from the superconductivity suppression due to the inverse proximity effect. Moreover, the s/F-multilayer structure acts as an additional source of superconductivity. However, the effective critical temperature of the magnetic superconductor is significantly smaller than of the bulk semiconductor material. This property of the system is demonstrated in Figure 3. Figure 3a presents the temperature dependence of the pair potential in different superconducting layers for P (solid lines) and AP (dashed) magnetization configurations in F-layers. In the large semiconductor electrode, the temperature dependence of the pair potential coincides with the prediction of the pure Bardeen–Cooper–Schrieffer (BCS) model. At the same time, the pair potential in thin s-layers rapidly increases near the effective critical temperature, *T*_C_* ≈ 0.5*T*_C_. It should be noticed that the superconductivity support from the bulk semiconductor source provides a nontrivial shape for Δ(*T*) in the closest s-layer, with a sharp increase in the pair potential to a constant value in the vicinity of *T*_C_*. The farther the layer, the weaker the support effect. Deep s-layers that are far from the bulk source are barely influenced by the bulk source and their properties are similar to independent (s/F)*_x_* multilayer structures with sloping Δ(*T*) dependencies. The spatial distributions of the Green’s functions (Figure 3b and Figure 3c) also have step-like shapes. Since the pair potential inside the multilayer structure has a superconducting source independent of the bulk semiconductor layer, the value of the pairing amplitude *F*_1_(*x*) is almost constant inside every s-layer. However, this value inside each layer is strongly dependent on the distance from the bulk electrode. At temperature values above *T*_C_*, the spatial distribution has a similar shape, although a significant pairing amplitude appears only in the s-layers closest to the bulk semiconductor electrode. An additional possible consequence of such spatial distribution appears in the screening of F-layers in multilayer structures from an outer magnetic field due to the Meissner effect. The inner F-layers are strongly screened while the opposite is observed for the outer layers. This means that the remagnetization of the layers in an increasing homogeneous external magnetic field do not occur simultaneously, but instead gradually from the outer to the inner layers of the structure.

The calculated distribution of the anomalous Green’s function, *F*, allows for the estimation of the screening properties of the hybrid structure. The spatial distribution of the screening length directly depends on the proximity of the superconducting order parameter in the system [25], given by


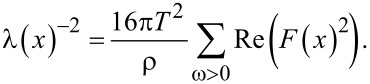


Hence, the screening length and the kinetic inductance of the considered s-layers are significantly higher in the P case compared to the AP case. This leads to a redistribution of the current flowing along the multilayer structure which increases the total kinetic inductance of the structure [26], according to the following relation


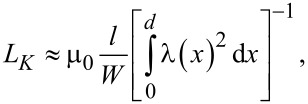


where *l* is the length of the strip, *W* and *d* are the width and the thickness of the multilayer structure, respectively. It can be concluded that small changes in the temperature or in the applied magnetic field [9] can significantly change (from zero to relatively large values) the kinetic inductance of thin s-layers in the hybrid structures studied in this work.

### Experimental results

The next step was to experimentally verify the significant changes, predicted in the model, for the pair potential in thin s-layers of a [Co(1.5 nm)/Nb(8 nm)/Co(2.5 nm)/Nb(8 nm)]_3_ AFM. For these superlattices, the possibility of switching between the P and AP cases, using a magnetic field with an intensity of ≈30 oersteds, has already been demonstrated [9]. The samples were prepared by using the magnetron sputtering system Leybold Heraeus Z-400 during a single deposition cycle without depressurization of the chamber. Only three targets were used for the structure preparation: niobium (99.95% purity) was used as a superconducting Cooper pair generator and interlayer separator between two neighboring films of ferromagnetic layers grown using cobalt (99.95% purity). Pure silicon (99.999%) was the third target used to create a passivating layer to prevent structure oxidation. The details regarding the deposition technology were previously described [27].

**Figure 4:**
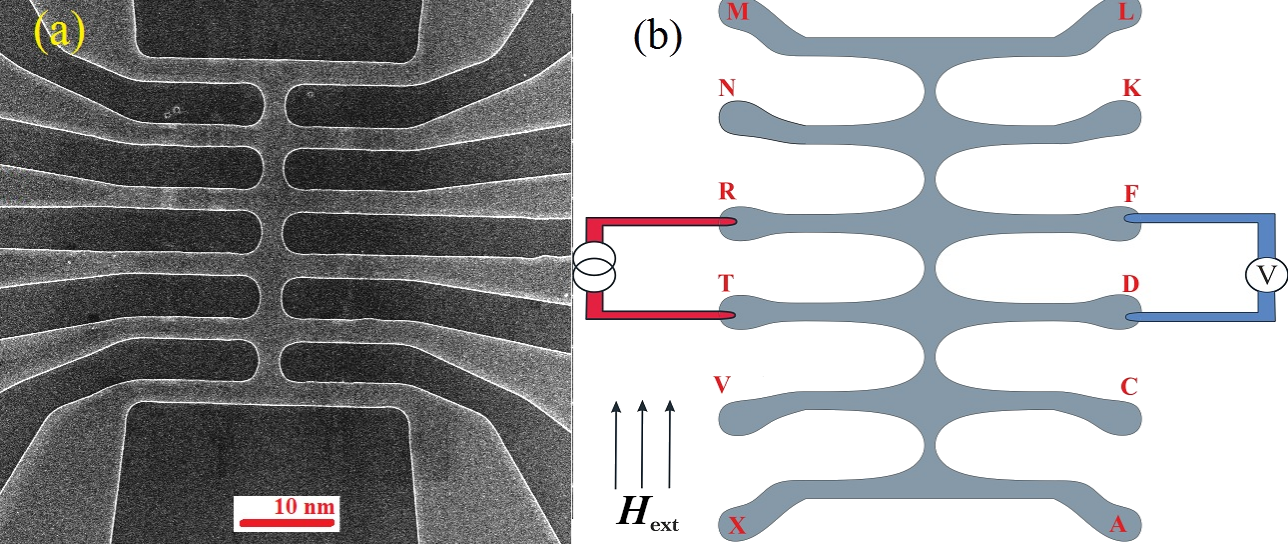
A micrograph (a) and a principal scheme (b) of the structure used for the critical temperature measurements (each contact was marked with a letter).

The structure for the transport measurements was etched under pure argon atmosphere (Ar^+^ milling) in a CRYO RIE Alba Nova machine (Stockholm University). The patterned design allowed for a four-point type measurement of six segments of the sample in one cooling cycle (Figure 4). The pair of contacts was applied for setting the current and the pair of microwires was used to test an induced potential difference. Each contact was marked with a letter. Therefore, each measurement was denoted by a pair of letters ("RT","TV", etc.). All the low-temperature measurements performed in this work were done using a cryogen-free magnet system with an insert for the flowing gas.

Figure 4 represents the principle scheme of the measurements performed in this work. This “centipede-like” sample design facilitates the measurements of the electrical resistance values in different synapse-like segments simply by alternating the arms. Before the measurements, the sample was cooled down to 10 K in a zero-field cooling mode and no current was applied. The critical temperature measurements started at 10 K and afterwards a temperature sweeping was performed (*R*(*T*) measurements). In addition, and external 1 μА current (AC mode with frequency of 127 Hz) was applied. The temperature change rate was chosen such that a minimal temperature gradient was generated in both downward and upward sweeping directions. Therefore, the resulting shapes of the curves resembled one another but with a slight 0.05 K shift.

**Figure 5:**
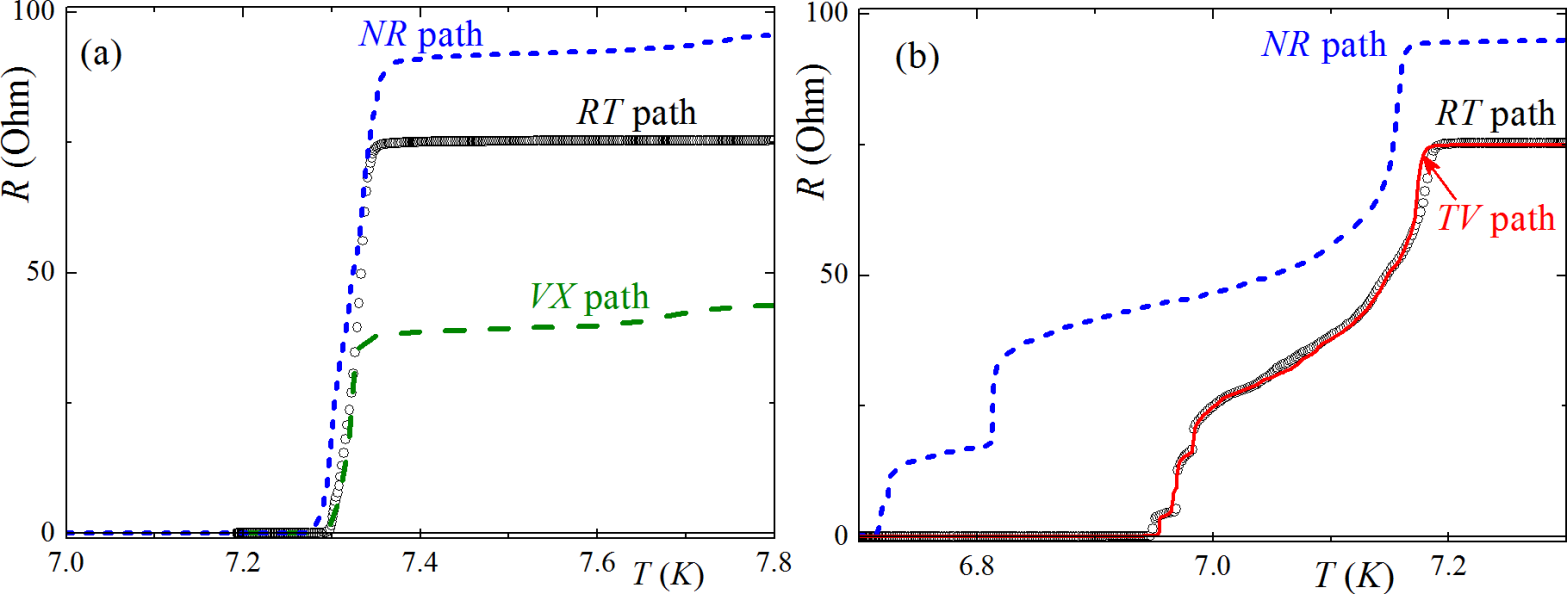
Resistance as a function of the temperature (a) without an initial magnetization of the sample, at a current *I* = 20 μA, and (b) after remagnetization in the longitudinal direction, at a current *I* = 1 mkA. Each contact in the “centipede-like” structure was marked with a letter. Therefore, each measurement was denoted by a pair of letters ("RT", "TV", etc.).

In this article, only the measurements performed in three segments were presented since the rest of the measurements showed a similar behavior. In the beginning of the experiment, the resistivity as a function of the temperature was measured without any applied external magnetic field for all the synapse-like segments, immediately after the sample was cooled down. In Figure 5a, the curves of the resistance, *R*, as a function of the temperature, *T*, are shown for the case in which there is no magnetic field applied. The results show a similarity to common homogeneous superconductors subjected to the same conditions: a slight change in the resistance is noticed above critical temperature and after that there is a quick decrease to zero in the critical temperature region at 7.3 K. Niobium enters a superconducting state within the entire volume of sample. The uppercase letters indicate a voltmeter contact connection in the principal scheme of the sample (Figure 4b). The current was applied to the opposite arm of the “centipede”. Note that, in the initial state, in which small domains in the Co layers were randomly distributed, resistive transitions (*R*(*T*)) at different segments (e.g., “RT” (black circles), “TV” (blue dashed line)), etc.) were similar in shape. The difference in the resistance values can be explained by geometrical factors, such as the shape and width of the segments.

The next series of measurements were carried out under the same conditions. However, for these experiments, the samples were previously submitted to a training protocol, in which they were exposed to an alternating magnetization in the field applied parallel to the layers of the sample at 10 K. The magnetic field ranged from −200 Oe to +200 Oe and, during this process, the electric current was not applied to the sample. After remagnetization, the *R*(*T*) behavior significantly changed. For all the segments the superconducting transition started at a lower temperature, *T* = 7.2 K, and the resistance gradually decreased until *T* = 6.7 K where it went to zero. Moreover, several distinct steps appeared in the *R*(*T*) curves, which we attribute to resistive transitions of individual superconducting layers (or groups of layers) in the multilayer structure. As explained previously in the simulations (Figure 2 and Figure 3), the effective critical temperature of the s-layers within the multilayer structure (i.e., the temperature in which the superconducting order parameter exceeds the value sufficient for carrying the applied transport current) depends on the magnetization orientation in the neighboring F-layers. It is at a maximum for the AP and at a minimum for the P orientation. From Figure 5b one can see that different segments may have a different sequence of steps. For example, the “RT” (black circles) and “TV” (red line) segments have a very similar *R*(*T*) curve, with three steps in the temperature range of 6.95 K < *T* < 7.05 K. Conversely, for the “NR” segment (blue dashed line) the steps have a different shape and expand down to *T* = 6.7 K. This variation indicates that, although the state of the multilayer structure is not homogeneous across the whole sample, it is homogeneous enough within each segment to cause a significant variation in the effective *T*_c_ values of individual layers with minimal values, corresponding to the P state.

Such behavior of *R*(*T*) in the vicinity of *T* = 7.3 K is similar to the behavior of *R*(*T*) in the S/N and S/F contacts upon the conversion of the current from normal to a supercurrent. Furthermore, there are several resistance jumps in the temperature region around *T* = 6.7 K. The actual number of jumps changes for different electrodes (Figure 5b).

The observed variation in the *R*(*T*) step-like behavior may also be due to the specific sample geometry (i.e., electrodes with horizontal and vertical orientations, Figure 4a). Due to the shape anisotropy, the “body” of the “centipede” is magnetized along the longitudinal direction, while the arms are perpendicular to the field. This geometry provides different magnetic structures and effective exchange fields for different parts of the structure. Probably the critical temperature of the arms is lower than in the body, providing the injection of a normal current into the body. The conversion process from normal to supercurrent provides the finite voltage, measured in the middle segments. Such measurement also gives some additional information about the properties of the arms. For instance, the dependence between *R*(*T*) and the current transport along the “RT*”* and “TV” (Figure 5b) segments are almost the same. This means that the source of the voltage is in the “T”-electrode, which is the source of the normal quasiparticles, while the “R”*-* and “V”*-*arms are in the superconductive state. At the same time, due to the connection between the “N”*-* and “R”*-*electrodes, the jumps occur at significantly smaller temperature values, which probably correspond to the resistive state of the “N”*-*electrode.

The results in Figure 5b reveal the presence of a series of jumps in the *R*(*T*) curves. The explanation for why the number of jumps exceeds two in the *R*(*T*) curve is beyond the trivial transition of electrodes to the superconducting state. There are two possible reasons for such behavior in this system. The first one is associated with the sequential transition of the thin s-layers in the middle part of the “centipede” that was measured. This mechanism is shown in Figure 3a. In that case, thin s-layers of the middle part of the structure transition, one by one, from a normal to a superconducting state, leading to a step-like change in the conversion rate of the current and to the appearance of steps in the *R*(*T*) curve. The steps in the “RT”*-* and “TV”*-*segments are suitable for that model, since they are close to each other. At the same time, the middle part of the “centipede”, between the connection of the electrodes “R”- and “V”-, stays in the single-domain state of the multilayer structure, while the part between the “N”- and “R”- electrodes stays in the other one.

The second possible explanation is based on the magnetic structure of the connected electrodes. If we assume that the “N”- (or “T”-) electrode consists of two (or three) domains with significantly different mutual magnetization orientation in the F-layers, this can provide different critical temperature values for each domain. Then, the transition of each domain to the superconducting state leads to the decrease in the resistance to the final value at the end of the experiment. This model can explain every jump distribution and how the *R*(*T*) curve behavior depends on the “RT”*-* and “TV”*-*segments. At the same time, the same model contradicts the presence of a thick S-bank at the bottom part of the electrode, which probably should determine its critical temperature, with a weak dependence on the magnetic state of the multilayer structure. Maybe, due to technical reasons, the S-bank becomes thinner in some arms, providing a stronger dependence of the critical temperature on a mutual magnetization. In the latter, both mechanisms can generate the steps on the *R*(*T*) curve.

## Discussion

We continued with the theoretical and experimental research on the Co/Nb multilayer structure since the polarized neutron reflectometry and superconducting quantum interference device (SQUID)-magnetometry results have proven that the effective exchange energy can be controlled by applying relatively weak magnetic fields. This time we focused on the "life" of superconductivity (pair potential) in thin s-layers in a changing magnetic environment.

The theoretical studies in the present article showed that it is possible to magnetically change the kinetic inductance of superconducting layers, and even transfer thin layers to a normal state at a fixed temperature.

The experimental results showed that the transition of thin s-layers to the normal state in the multilayer structure is possible. In addition, the temperature of this transition depends on the magnetic environment.

Given the aforementioned results, one can conclude that the electronic transport properties in the multilayer structure S/[F1/s/F2/s]*_n_* can be used to create different switching electronic elements, including synapses. This new type of application will be discussed in more detail.

The creation of artificial neural networks is one of the current trends in the development of superconductor electronics [10–15]. Such an artificial neural network contains layers of elements that nonlinearly transform the incoming signal (neurons), which is connected by linear tunable connections (synapses). There are more than 10^6^ synapses in the neural networks that are used in these applications. The energy dissipation at these interconnects is a serious problem, which motivates the search for energy-efficient superconducting solutions in this research field.

In the early 1990s, two-contact interferometers and their modifications, operating in a resistive mode, were used as basic elements (artificial neurons) in a superconducting ANN. In these neurons, the signal level usually corresponded to the average voltage level in the cell. As a result, these ANN schemes were neither fast nor energy efficient. New studies in this area appeared again at the end of the 2000s. This revival was associated with the spike in neural networks, in which the information is presented as a sequence of identical (single-quantum) voltage pulses, and the signal corresponds to a time delay between pulses [10,21]. From a circuitry point of view, such neural networks resemble the rapid single flux quantum (RSFQ) logic devices. Therefore, the development of energy-efficient RSFQ logic devices motivated us to investigate neural networks (and primarily synapses) with an ultra-small energy dissipation. That was done based on adiabatic superconducting logic cells with the presentation of information in the form of magnitudes and directions of currents in the superconducting circuits [17–19].

The main problem in these approaches is the complexity of the practical implementation of an effective synaptic connection: they should be tunable but, at the same time, retain a memory effect. In order to create a synapse, a Josephson contact with a ferromagnetic component in the weak coupling region was recently proposed to adjust the critical current during the functioning of a neural network [20].

In this paper, we propose a way to eliminate completely the Josephson nonlinearity from the synapse circuit. The processes of switching on and off the superconductivity in the thin s-layers, surrounded by magnetic materials, can be used to vary the transmission coefficient of the simplest synapse, shown in Figure 1a. The dependence of the output current of the inductive synapse, *i*_out_, with the parasitic inductance, *l_p_*, and the sum of the controlled kinetic inductors of the arms, Σ*l*, is presented below. The configurable dynamic range of the element increases with the difference between the kinetic inductances of the arms and decreases with the rise of the geometric inductance.

**Figure 6:**
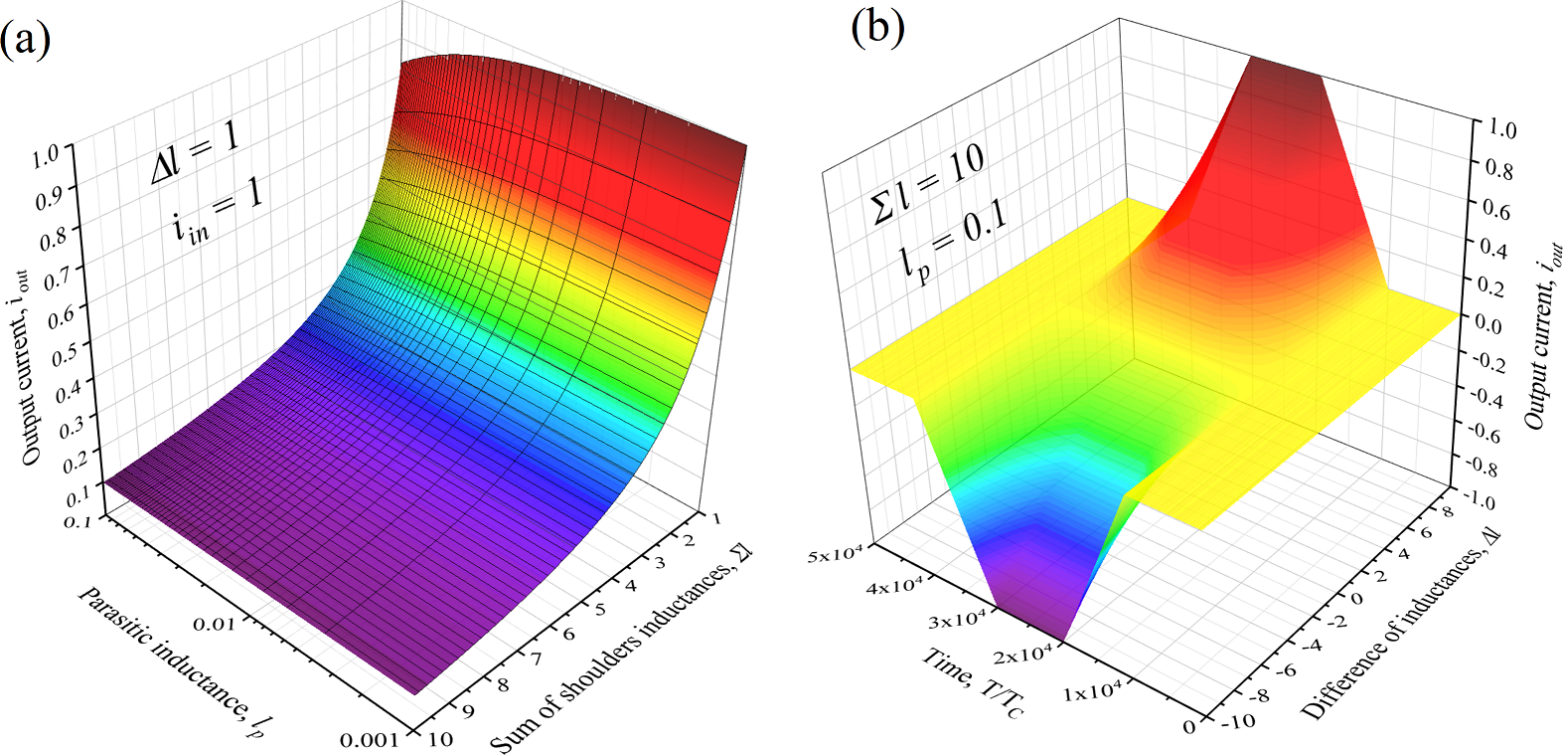
(a) The output current, *i_out_*, of the inductive synapse versus the geometric inductance, *l_p_*, and the sum of the controlled kinetic inductors of the arms, Σ*l*. (b) The output current under the action of a single input pulse for different values of the difference between the inductors of the arms, Δ*l*.

Taking into account more complex physical phenomena in the S/F multilayer structure, the future studies aim to increase the functionality of the proposed synapse. With a noncollinear magnetization in the neighboring Co layers, the formation of a long triplet component in a supercurrent is possible in the considered AFM [28–31]. This allows for a significant influence on the magnitude of the order parameter in superconducting layers, controlling the misorientation in the magnetization angles. The triplet superconducting correlations of electrons are formed from singlet correlations. Hence, this effect reduces the kinetic inductance of the s-layers in a quasi-monotonic manner with the magnitude of the controlling magnetic field. This effect enables the construction of a magnetically tunable kinetic inductor for artificial neural networks.

## References

[1] Baek B, Rippard W H, Benz S P, Russek S E, Dresselhaus P D (2014). Nat Commun.

[2] Alidoust M, Halterman K (2014). Phys Rev B.

[3] Gingrich E C, Niedzielski B M, Glick J A, Wang Y, Miller D L, Loloee R, Pratt W P, Birge N O (2016). Nat Phys.

[4] Shafranjuk S, Nevirkovets I P, Mukhanov O A, Ketterson J B (2016). Phys Rev Appl.

[5] Soloviev I I, Klenov N V, Bakurskiy S V, Kupriyanov M Y, Gudkov A L, Sidorenko A S (2017). Beilstein J Nanotechnol.

[6] Shafraniuk S E, Nevirkovets I P, Mukhanov O A (2019). Phys Rev Appl.

[7] Golod T, Kapran O M, Krasnov V M (2019). Phys Rev Appl.

[8] Satchell N, Shepley P M, Algarni M, Vaughan M, Darwin E, Ali M, Rosamond M C, Chen L, Linfield E H, Hickey B J (2020). Appl Phys Lett.

[9] Klenov N, Khaydukov Y, Bakurskiy S, Morari R, Soloviev I, Boian V, Keller T, Kupriyanov M, Sidorenko A, Keimer B (2019). Beilstein J Nanotechnol.

[10] Schneider M L, Donnelly C A, Russek S E (2018). J Appl Phys.

[11] Harada Y, Goto E (1991). IEEE Trans Magn.

[12] Hidaka M, Akers L A (1991). Supercond Sci Technol.

[13] Mizugaki Y, Nakajima K, Sawada Y, Yamashita T (1993). Appl Phys Lett.

[14] Mizugaki Y, Nakajima K, Sawada Y, Yamashita T (1994). IEEE Trans Appl Supercond.

[15] Crotty P, Schult D, Segall K (2010). Phys Rev E.

[16] Chiarello F, Carelli P, Castellano M G, Torrioli G (2013). Supercond Sci Technol.

[17] Yamanashi Y, Umeda K, Yoshikawa N (2013). IEEE Trans Appl Supercond.

[18] Schegolev A E, Klenov N V, Soloviev I I, Tereshonok M V (2016). Beilstein J Nanotechnol.

[19] Klenov N V, Schegolev A E, Soloviev I I, Bakurskiy S V, Tereshonok M V (2018). IEEE Trans Appl Supercond.

[20] Soloviev I I, Schegolev A E, Klenov N V, Bakurskiy S V, Kupriyanov M Y, Tereshonok M V, Shadrin A V, Stolyarov V S, Golubov A A (2018). J Appl Phys.

[21] Schneider M L, Donnelly C A, Russek S E, Baek B, Pufall M R, Hopkins P F, Dresselhaus P D, Benz S P, Rippard W H (2018). Sci Adv.

[22] Usadel K D (1970). Phys Rev Lett.

[23] Kuprianov M Y, Lukichev V F (1988). Sov Phys - JETP.

[24] Bakurskiy S V, Kupriyanov M Y, Baranov A A, Golubov A A, Klenov N V, Soloviev I I (2015). JETP Lett.

[25] Mironov S, Mel'nikov A S, Buzdin A (2018). Appl Phys Lett.

[26] Annunziata A J (2010). Single-photon detection, kinetic inductance, and non-equilibrium dynamics in niobium and niobium nitride superconducting nanowires.

[27] Morari R, Zdravkov V, Antropov E, Sidorenko A (2012). J Nanoelectron Optoelectron.

[28] Bergeret F S, Volkov A F, Efetov K B (2001). Phys Rev Lett.

[29] Houzet M, Buzdin A I (2007). Phys Rev B.

[30] Niedzielski B M, Diesch S G, Gingrich E C, Wang Y, Loloee R, Pratt W P, Birge N O (2014). IEEE Trans Appl Supercond.

[31] Zdravkov V I, Lenk D, Morari R, Ullrich A, Obermeier G, Müller C, Krug von Nidda H-A, Sidorenko A S, Horn S, Tidecks R (2013). Appl Phys Lett.

